# l-phenylalanine modulates gut hormone release and glucose tolerance, and suppresses food intake through the calcium-sensing receptor in rodents

**DOI:** 10.1038/ijo.2017.164

**Published:** 2017-08-08

**Authors:** A Alamshah, E Spreckley, M Norton, J S Kinsey-Jones, A Amin, A Ramgulam, Y Cao, R Johnson, K Saleh, E Akalestou, Z Malik, N Gonzalez-Abuin, A Jomard, R Amarsi, A Moolla, P R Sargent, G W Gray, S R Bloom, K G Murphy

**Affiliations:** 1 Section of Endocrinology and Investigative Medicine, Department of Medicine, Hammersmith Hospital, Imperial College London, London, UK; 2 Oxford Centre for Diabetes, Endocrinology and Metabolism, University of Oxford, Oxford, UK; 3 TasteTech Ltd, Bristol, UK

## Abstract

**Objective:**

High-protein diets (HPDs) are associated with greater satiety and weight loss than diets rich in other macronutrients. The exact mechanisms by which HPDs exert their effects are unclear. However, evidence suggests that the sensing of amino acids produced as a result of protein digestion may have a role in appetite regulation and satiety. We investigated the effects of l-phenylalanine (L-Phe) on food intake and glucose homeostasis in rodents.

**Methods:**

We investigated the effects of the aromatic amino-acid and calcium-sensing receptor (CaSR) agonist l-phenylalanine (L-Phe) on food intake and the release of the gastrointestinal (GI) hormones peptide YY (PYY), glucagon-like peptide-1 (GLP-1) and ghrelin in rodents, and the role of the CaSR in mediating these effects *in vitro* and *in vivo.* We also examined the effect of oral l-Phe administration on glucose tolerance in rats.

**Results:**

Oral administration of l-Phe acutely reduced food intake in rats and mice, and chronically reduced food intake and body weight in diet-induced obese mice. Ileal l-Phe also reduced food intake in rats. l-Phe stimulated GLP-1 and PYY release, and reduced plasma ghrelin, and also stimulated insulin release and improved glucose tolerance in rats. Pharmacological blockade of the CaSR attenuated the anorectic effect of intra-ileal l-Phe in rats, and l-Phe-induced GLP-1 release from STC-1 and primary L cells was attenuated by CaSR blockade.

**Conclusions:**

l-Phe reduced food intake, stimulated GLP-1 and PYY release, and reduced plasma ghrelin in rodents. Our data provide evidence that the anorectic effects of l-Phe are mediated via the CaSR, and suggest that l-Phe and the CaSR system in the GI tract may have therapeutic utility in the treatment of obesity and diabetes. Further work is required to determine the physiological role of the CaSR in protein sensing in the gut, and the role of this system in humans.

## Introduction

Obesity is a major health problem associated with serious comorbidities, including type II diabetes.^1, 2^ Diet and life style modifications are often difficult to adhere to and thus ineffective over long periods. High-protein diets suppress food intake and reduce weight gain or increase weight loss.^3, 4^ The expression of the promiscuous amino-acid-sensing receptors, the umami taste receptor T1R1/T1R3, the G-protein-coupled receptor family C group 6 member A and the calcium-sensing receptor (CaSR)^5, 6^ in the gastrointestinal (GI) tract has led to the suggestion that they may sense to the amino-acid products of protein digestion to mediate satiety.^6^ There is *in vitro* evidence that these receptors may be involved in gut nutrient sensing,^7, 8, 9^ but their role in nutrient sensing and satiety *in vivo* has been little investigated.^10^ The CaSR is named for its well-characterised role in calcium homeostasis, but in fact this receptor regulates a number of cellular processes, including cell proliferation and differentiation.^11, 12, 13^ Calcium is the major activating ligand, but the CaSR also allosterically binds aromatic l-amino acids and, less potently, aliphatic amino acids.^14, 15^

l-Phenylalanine (L-Phe) is a proteinogenic and essential aromatic amino acid derived from dietary intake and the recycling of amino-acid stores in the body.^16^ l-Phe is utilised in protein synthesis, tyrosine synthesis and as a substrate in other biochemical pathways.^17^ l-Phe is the most potent amino-acid activator of the CaSR.^18^ The CaSR is expressed on hormone-secreting enteroendocrine cells, leading to suggestions that it may sense amino acids within the GI tract to modulate gut hormone release.^6, 19^ l-Phe stimulates the release of cholecystokinin (CCK) from STC-1 cells,^20^ and l-Phe and l-tryptophan stimulate CCK secretion from isolated I-cells.^21^ In addition, specific l-amino acids, including l-Phe, stimulate the release of the energy and glucose homeostasis-regulating gut hormones peptide YY (PYY), glucagon-like peptide 1 (GLP-1) and gastric inhibitory polypeptide from isolated rat small intestine through a CaSR-dependent mechanism.^22^ However, to date, the role of CaSR in hormone release, or glucose or energy homeostasis has not been studied *in vivo*. We thus investigated the effects of l-Phe on food intake and body weight, energy expenditure, gut hormone release and glucose tolerance, and the role of the CaSR in mediating these effects in rodents.

## Materials and methods

### Animals

Male Wistar rats (200–250 g; Charles River, Margate, UK) and male C57BL/6 mice, 8–10 weeks (Harlan, Bicester, UK) were individually housed under controlled temperature (21–23 °C) and humidity on a 12 h light:12 h darkness cycle. All animals had *ad libitum* access to standard chow RM1 (SDS, Witham, UK) and water unless stated otherwise. All animal procedures were in accordance with the UK Home Office Animals (Scientific Procedures) Act 1986 and approved by the Animal Welfare and Ethics Review Board at the Central Biological Services unit at the Hammersmith Campus of Imperial College London.

### Feeding studies

Rats and mice were acclimatised to the experimental procedures and randomised by body weight. Rats and mice were fasted for 16 h overnight and subsequently received an oral gavage (OG; 3 ml) of vehicle (10% TWEEN20 in water) or l-Phe (Sigma, Poole, UK) at doses stated in the results in the early hours of the light phase. For dark-phase-feeding studies, rats and mice had *ad libitum* access to food and were administered agents at the onset of dark phase. For intra-ileal administration studies, rats received 0.5 ml of vehicle or 10 mm l-Phe; a concentration previously shown to stimulate GLP-1 and PYY release from isolated rat intestinal loops^22^ and of a similar order of magnitude to the levels of l-Phe in the ileum following protein consumption.^23^ For the CaSR agonist R658 hydrochloride (R568.HCl) study, overnight-fasted rats received an OG of vehicle (1.8% dimethyl sulfoxide in water) or 1.1 mg kg^−1^ of R568.HCl (TOCRIS Bioscience, Bristol, UK; *n*=8, vehicle; 9, R568) during the early light phase. This dose of R568.HCl has previously been shown not to effect systemic calcium homeostasis.^24^ Rats and mice were returned to their cages immediately following administration with pre-weighed amounts of food and food intake measured at 1, 2, 4, 8 and 24 h following administration.

The effect of intra-ileal administration of l-Phe was investigated in conscious rats. Male Wistar rats weighing 250–300 g were implanted with a catheter into the ileum, ~5 cm from the ileocaecal junction. The catheter was tunnelled to the scapular region and secured in place using a vascular access harness (Instech Laboratories Inc., Plymouth Meeting, PA, USA). The surgery was performed by Charles River (Charles River, Margate, UK) and rats were transferred to Imperial College before experimentation.

Having established the anorectic effect of intra-ileal l-Phe administration and the expression of CaSR within GI tract, the role of the CaSR in mediating this effect was examined using pharmacological blockade of the receptor in rats. The CaSR antagonist NPS2143 hydrochloride (NPS2143.HCl) was co-administered with l-Phe directly into the ileum to specifically target the CaSR population within the ileum where both GLP-1 and PYY are also highly expressed.^19^ This allowed us to know that both agents were concurrently present in the same region of the gut, and to utilise a low dose of NPS2143.HCl unlikely to alter systemic calcium homeostasis. Rats with *ad libitum* access to food were injected directly into the ileum via the catheter access port at the beginning of dark cycle with vehicle (0.5% dimethyl sulfoxide and 0.9% saline), 1 μm of the CaSR antagonist NPS2143.HCl, 10 mm l-Phe or 10 mm l-Phe in combination with 1 μm NPS2143.HCl (*n*=18, crossover), in a total injection volume of 0.5 ml. Rats were immediately returned to their cages and food intake measured at 1, 2, 4, 8 and 24 h post administration.

### Energy expenditure studies

Rats were individually placed in a 24-chamber open-circuit comprehensive laboratory animal-monitoring system (Columbus Instruments, Columbus, OH, USA) and acclimatised to the system for 24 h. *Ad libitum*-fed rats received an OG of vehicle (10% TWEEN20 in water) or 6 mmol kg^−^^1^ l-Phe (*n*=8) at 1900 hours (early dark phase). VO_2_ and VCO_2_ were measured, and respiratory exchange ratio calculated by determining the ratio between CO_2_ produced/O_2_ and normalising the values to body weight. Ambulatory activity was simultaneously recorded by recording consecutive photobeam breaks.^25^ All recordings were taken every 16 min for 12 h following treatment administration. High-resolution food intake data were recorded every minute for a period of 120 min following oral administration.

### Murine colonic crypt isolation and secretion assays

Primary mice colonic crypt isolation and secretion studies were performed by adapting an established method previously described.^26, 27^ STC-1 cells were obtained from the American Type Culture Collection and maintained and cultured, and secretion experiments performed, as previously described.^20, 27^ They tested negative for mycoplasma contamination.

### Murine ileal organoid culture and secretion assays

Intestinal crypts from the ileums of 6- to 12-week-old male C57BL/6 mice were isolated, cultured and grown into organoids as previously described.^28^ Crypts were seeded on 48-well plates in 25 μl droplets per well comprising a mix of 12.5 μl Matrigel (Cultrex BME 2 RGF (ORGANOID MATRIX) PathClear) (Amsbio, Abingdon, UK) and 12.5 μl complete growth medium, comprised of Advanced DMEM/F12 (containing Primocin, 1 m HEPES and Glutamax (100 ×) (Invitrogen, Paisley, UK)), supplements N2 (100 ×), B27 (50 ×) (Invitrogen) and 500 mm *N*-acetylcysteine (Sigma-Aldrich, Poole, UK), and the following growth factors: 100 ng ml^−1^ mouse Noggin (Peprotech, London, UK), 50 ng ml^−1^ mouse epidermal growth factor (Invitrogen), 3 μm CHIR99021 (Sigma-Aldrich) and 10% R-spondin-1 conditioned medium. Crypt droplets were cultured in 300 μl complete growth medium in a 5% CO_2_ humidified atmosphere at 37 °C. Medium was changed every 2–3 days. To promote stem cell differentiation, CHIR99021 was removed from the medium 3 days after passaging. Mouse ileum organoids were passaged every 5–7 days at a 1:2–1:3 split ratio using Gentle Cell Dissociation Reagent (STEMCELL Technologies, Cambridge, UK) and mechanical disruption.

Treatments were administered when the organoids were at passage 4. On day 3 after splitting, CHIR99021 was removed and organoids incubated (37 °C, 5% CO2) in growth medium or growth medium containing 30 mm l-Phe or 15 μm NPS2390, or a combination of the two for 24 h. Supernatants were collected and wells treated with 50 μl lysis buffer and frozen at −20 °C overnight. Each well was washed with 50 μl Advanced DMEM/F12 and the contents scraped and collected. All lysates and supernatants were stored at −20 °C before analysis.

### Glucose tolerance test

Intraperitoneal glucose tolerance test (IPGTT) was performed following OG of l-Phe in rats. Rats were fasted overnight before receiving an intraperitoneal injection of 20% glucose (2 g kg^−1^) followed by an immediate OG of vehicle (10% TWEEN20 in water) or 6 mmol kg^−1^ l-Phe (*n*=15). A dose of 6 mmol kg^−1^ was used as it resulted in a greater effect on food intake than 3 mmol kg^−1^ in *ad libitum*-fed rats. Blood samples were taken from the tail vein immediately before glucose injection (*t*=0) and at 15, 30, 60, 90 and 120 min following administration, and blood glucose measured using a 65 glucometer (Contour meter and test strips, Bayer, Berkshire, UK). Insulin levels were measured at 30 min.

### GI hormone studies in rats

Rats were fasted overnight and received an OG of either water or 3 mmol kg^−1^ l-Phe (*n*=11), and were immediately returned to their cages. Rats were decapitated 30 min following administration. For cannulated rats, 150 μl of blood was collected via the jugular vein catheter 15 min following direct ileal administration of 0.5 ml of 10 mm l-Phe. Plasma was separated and stored as previously described.^27^

### Chronic feeding studies

Male mice 6–8 weeks of age were grouped-housed (*n*=5 per cage) with *ad libitum* access to water and 60% high-fat diet (Research Diets, New Brunswick, NJ, USA) for 8 weeks. Mice were then individually housed and given 1 week to acclimatise before experiments. Mice received an OG of vehicle (10% TWEEN20 and water) or 12 mmol kg^−1^ l-Phe twice throughout each dark phase for four nights, and three times for each of the three subsequent nights (*n*=10). Body weight and food intake were measured daily at the beginning of dark phase and 1 h following the first daily gavage. Mice were decapitated 24 h following the last administration of l-Phe and plasma collected and stored as described above.

### Hormone measurement

Rat/mouse active ghrelin enzyme-linked immunosorbent assay, insulin radioimmunoassay, leptin enzyme-linked immunosorbent assay (Millipore, Darmstadt, Germany) and CCK enzyme immunoassay kits (Sigma, Poole, UK) were used according to the manufacturers’ instructions. GLP-1 and PYY were measured using previously described in-house radioimmunoassays.^29, 30^

### Statistical analysis

All data are expressed as mean±s.e.m.’s and area under the curve (AUC), where appropriate. Studies were powered based on previous studies to detect 20% differences in food intake and GLP-1 levels, and 15% differences in energy expenditure or glucose AUC. Animals were randomised by body weight for feeding studies, and the data analysed using *T*-test or one-way analysis of variance (ANOVA) with Tukey’s *post hoc* test, where appropriate. Investigators were blinded to treatment until data analysis. Energy expenditure and cumulative food intake data were analysed using Šídák’s multiple comparison test with a repeated-measures ANOVA, and the AUC by *T*-test. All *in vitro* studies were analysed using one-way ANOVA with Dunnett’s test with exception of the CaSR antagonist secretion study, for which one-way ANOVA and Tukey’s *post hoc* test were applied. Gut hormone studies *in vivo*, glucose AUC and insulin levels were analysed using *T*-test. Chronic studies and the glucose tolerance test were analysed using two-way ANOVA with Sidak’s *post hoc* test. Animals were excluded from analysis if data was not within two s.d.’s of the mean. *P*<0.05 was considered statistically significant. All analysis was carried out using Graphpad Prism software (Prism 6.03, GraphPad Software Inc., San Diego, CA, USA).

## Results

### l-Phe reduces food intake in rodents

**Figure 1:**
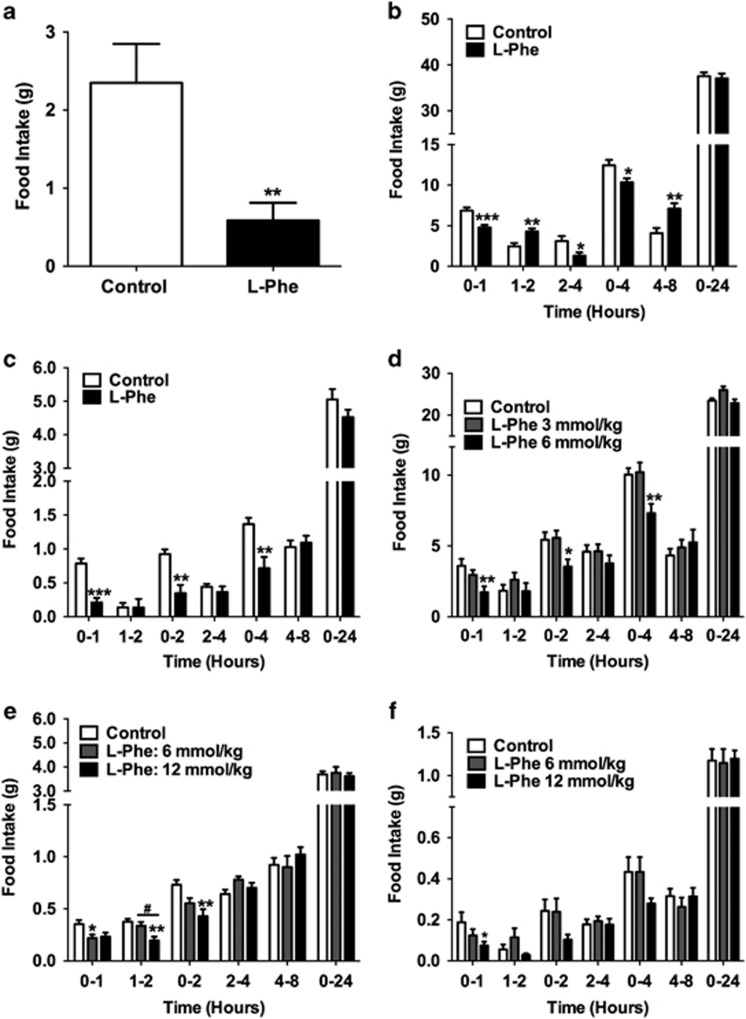
The effect of l-Phe on food intake in rodents. (**a**) The effect of intra-ileal administration of 10 mm l-Phe on food intake in *ad libitum*-fed rats at 0–1 h post administration during early dark phase (*n*=9 crossover, *P*<0.01 vs control). The effect of OG of control (water) and 3 mmol kg^−1^ l-Phe on food intake in (**b**) male rats following an overnight fast (*n*=13, vehicle; 11, L-Phe; *P<0.05, **P<0.01, ***P<0.001 vs control) and in (**c**) fasted male mice following an overnight fast receiving an OG of vehicle (10% TWEEN20 in water) and 12 mmol kg^−1^ l-Phe (*n*=5, **P<0.01, ***P<0.001 vs control). (**d**) The effect of OG of vehicle (10% TWEEN20 in water), and 3 and 6 mmol kg^−1^ l-Phe in *ad libitum*-fed rats at the beginning of dark phase (*n*=10, *P<0.05, **P<0.01; 6 mmol kg^−1^ l-Phe vs control). (**e**) The effect of OG of vehicle (10% TWEEN20 in water), and 6 and 12 mmol kg^−1^ l-Phe in *ad libitum* chow-fed mice at the beginning of dark phase (*n*=10; *P<0.05, 6 mmol kg^−1^ l-Phe vs control; **P<0.01, 12 mmol kg^−1^ l-Phe vs control; ^#^*P*<0.05, 6 vs 12 mmol kg^−1^ l-Phe), and in (**f**) *ad libitum* high-fat diet-fed DIO mice (*n*=10; *P<0.05, 12 mmol kg^−1^ l-Phe vs control). All figures show food intake at 0–1, 1–2, 0–2 (except **a**), 2–4, 4–8 and 0–24 h post administration. All data presented as mean±s.e.m.

We investigated the anorectic effect of intra-ileal and oral l-Phe administration in rodents. Both GLP-1 and PYY are highly expressed in the ileum, and the ileum may have a role in the greater satiety observed following a high-protein meal. Intra-ileal administration of 10 mm l-Phe significantly reduced food intake in *ad libitum*-fed rats during the 0–1 h interval during dark phase (*P*<0.01, *n*=9, crossover; Figure 1a). Furthermore, oral administration of l-Phe significantly decreased food intake in both rats and mice (Figures 1b and f). In fasted rats, OG of 3 mmol kg^−1^ l-Phe significantly reduced food intake compared to the control at 0–1, 2–4 and 0–4 h intervals following administration in the light phase (*n*=13, vehicle; 11, l-Phe; Figure 1b). OG of 6 mmol kg^−1^ l-Phe significantly reduced food intake in *ad libitum*-fed rats 0–1, 0–2 and 0–4 h following administration at the onset of the dark phase (*n*=10; Figure 1d). Similarly, OG of 12 mmol kg^−1^ l-Phe in fasted mice significantly reduced cumulative food intake 0–1, 0–2 and 0–4 h following administration during the light phase compared to vehicle (*n*=5; Figure 1c). In *ad libitum*-fed mice, OG of 6 mmol kg^−1^ l-Phe significantly reduced food intake in mice 0–1, 1–2 and 0–2 h post administration in the dark phase (*n*=10; Figure 1e). In addition, OG of 12 mmol kg^−1^ l-Phe reduced food intake in diet-induced obese (DIO) mice during 0–1 h period post administration (*P*<0.01, *n*=10; Figure 1f).

### l-Phe increases energy expenditure in rats

**Figure 2:**
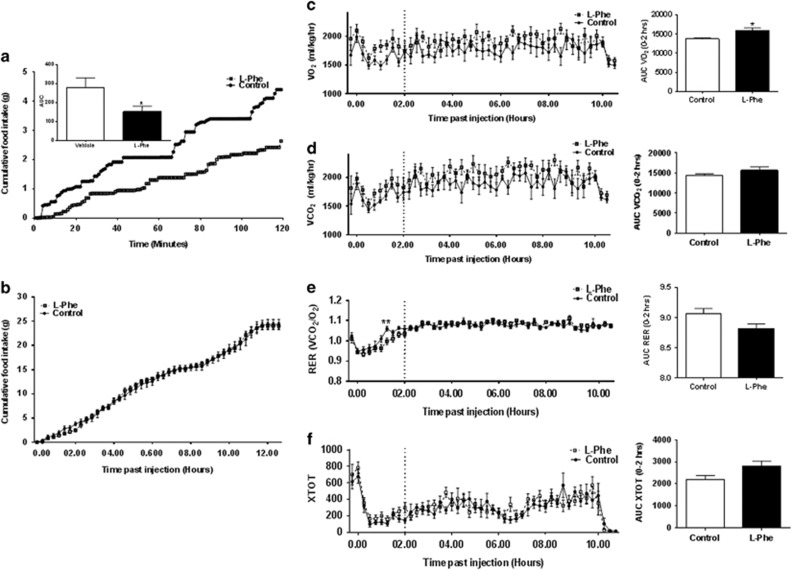
The effect of oral administration of l-Phe on food intake, energy expenditure and activity in rats. The effect of OG administration of vehicle (10% TWEEN20 in water; control) or 6 mmol kg^−1^ l-Phe on (**a**) the timeline of cumulative food intake and the AUC at 0–120 min, (**b**) the timeline of cumulative food intake at 0–12 h, (**c**) the timeline of VO_2_ and the AUC at 0–120 min; (**d**) the timeline of VCO_2_ and the AUC at 0–120 min; (**e**) the timeline of respiratory exchange ratio (RER) and the AUC at 0–120 min, (**f**) the timeline of activity (XTOT) and the AUC at 0–120 min in rats injected at the onset of the dark phase and placed in comprehensive laboratory animal-monitoring system cages for 12 h. Recordings were taken over a period of 12 h at 16 min intervals following administration. High-resolution food intake recordings were taken every minute for 120 min. Dotted lines on graphs represent the 0–120 min interval. Data presented as mean±s.e.m. and AUC. *n*=8, *P<0.05.

OG of 6 mmol kg^−1^ l-Phe reduced food intake in rats in comprehensive laboratory animal-monitoring system metabolic cages during the 0–120 min interval (AUC: *P*<0.05 vs control, *n*=8; Figure 2a), though without effect at later time points (Figure 2b). OG of 6 mmol kg^−1^ l-Phe increased the maximal oxygen consumption (VO_2_) and the maximal carbon dioxide production (VCO_2_); this effect achieved statistical significance for VO_2_ during the 0–120 min interval following administration (AUC VO_2_: *P*<0.05 vs control; Figures 2c and d). Respiratory exchange ratio was also lower in l-Phe-treated rats during 0–120 min following administration (Figure 2e). OG of 6 mmol kg^−1^ l-Phe had no significant effect on locomotor activity (Figure 2f).

### l-Phe stimulates GI hormone release

**Figure 3:**
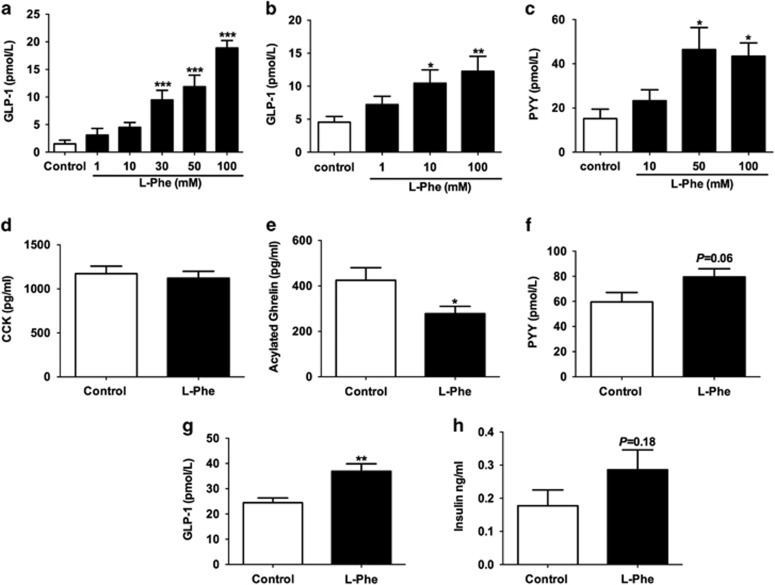
The effect of l-Phe on gut hormone release. (**a**) The effect of 1, 10, 30, 50 and 100 mm l-Phe on GLP-1 release from STC-1 cells. Cells were incubated with each treatment for 2 h. *n*=9 independent plates. *P<0.05, **P<0.01, P***<0.001 vs control. (**b**) The effect of l-Phe on GLP-1 release from primary mice colonic L cells incubated with 1, 10 and 100 mm l-Phe for 2 h. *n*=6 plates from 6 mice; and (**c**) the effect of l-Phe on PYY release from primary mice colonic L cells incubated with 10, 50 and 100 mm l-Phe for 2 h. *n*=9 plates from 9 mice. *P<0.05, **P<0.01 vs control. The effect of OG of water (control) or 3 mmol kg^−1^ l-Phe on (**d**) plasma CCK and (**e**) plasma acylated ghrelin 30 min after administration in rats following an overnight fast (*n*=11, *P<0.05 vs control). (**f**) The effect of intra-ileal administration of 10 mm l-Phe on PYY levels 15 min following administration in overnight-fasted rats (*n*=7, vehicle; 10, L-Phe; *P*=0.06 vs control). The effect of OG of water (control) or 3 mmol kg^−1^ l-Phe on (**g**) plasma GLP-1 and (**h**) plasma insulin 30 min after administration in rats following an overnight fast (*n*=11, **P<0.01 vs control).

l-Phe (30 mm) increased GLP-1 release by ~9-fold from STC-1 cells (*n*=9 independent plates; Figure 3a), and 10 and 100 mm l-Phe significantly stimulated the release of GLP-1 from a murine primary colonic L-cell culture (*n*=6 independent plates from 6 mice; Figure 3b). Similarly, 50 and 100 mm l-Phe significantly stimulated PYY release (*n*=9 independent plates from 9 mice; Figure 3c).

Oral l-Phe administration of 3 mmol kg^−1^ in rats (a dose shown to suppress appetite in fasted rats) significantly increased plasma GLP-1 levels (*n*=11 per group; Figure 3g), and resulted in a non-significant (*P*=0.18) rise in insulin levels (Figure 3h), but had no effect on plasma CCK levels at this time point (Figure 3d). However, OG of l-Phe significantly reduced plasma acylated ghrelin levels (*n*=11 per group; Figure 3e). Furthermore, direct intra-ileal administration of 10 mm l-Phe appeared to increase plasma PYY levels in conscious rats, though this effect did not achieve statistical significance (*P*=0.07, *n*=7, vehicle; 10, l-Phe; Figure 3f).

### l-Phe improves glucose tolerance in rats

**Figure 4:**
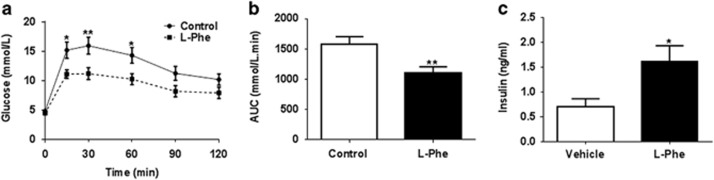
The effect of l-Phe on glucose homeostasis in rats. The effect of oral l-Phe on (**a**) glucose tolerance test and (**b**) AUC for glucose in overnight-fasted male rats that received an intraperitoneal injection of 20% glucose solution (2 g kg^−1^ body weight) followed by an immediate OG of 6 mmol kg^−^^1^ l-Phe. (**c**) Plasma insulin levels measured at *t*=30 during IPGTT (*n*=15, *P<0.05, **P<0.01 vs control). All data are presented as mean±s.e.m. and AUC.

GLP-1 is a potent incretin.^31^ Having established the ability of oral l-Phe to increase plasma GLP-1 levels in rats, we hypothesised that this effect would improve glucose tolerance, and carried out an IPGTT in rats that received an oral dose of 6 mmol kg^−1^ l-Phe. An IPGTT was used rather than an oral glucose tolerance test as it was felt that any effect of increased incretin release would be better observed using an IPGTT. OG of l-Phe significantly lowered plasma glucose levels following an IPGTT in rats (l-Phe AUC: *P*<0.01 vs control, *n*=15; Figures 4a and b). Furthermore, plasma insulin levels were significantly higher at 30 min following IPGTT in rats orally gavaged with l-Phe (*P*<0.05, *n*=15; Figure 4c).

### Specific effects of l-Phe on food intake and gut hormone release are mediated via CaSR

**Figure 5:**
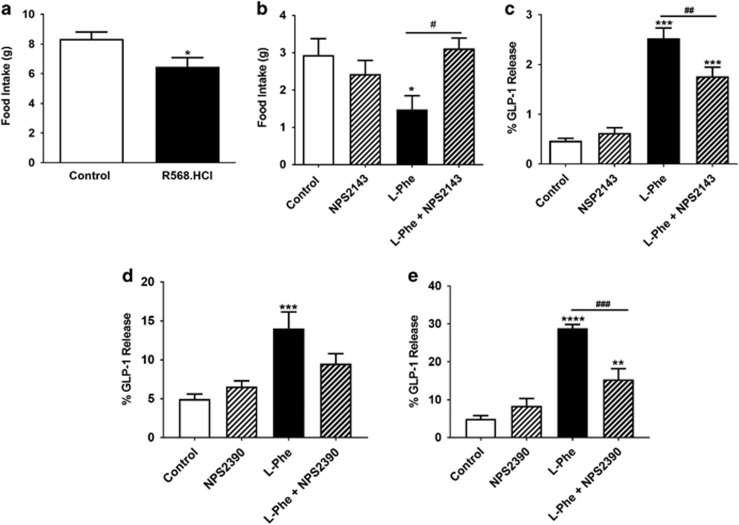
The role of CaSR in mediating the anorectic effect of l-Phe. (**a**) The effect of oral administration vehicle (1.85% dimethyl sulfoxide (DMSO) in water) and 1.1 mg kg^−1^ R568.HCl in overnight-fasted rats at 0–1 h following administration during early hours of light phase (*n*=8, vehicle; 9, L-Phe; *P*<0.05 vs control); and (**b**) the effect of intra-ileal administration of vehicle (0.25 DMSO, 0.9% saline), 1 μm NPS.2143.HCl, 10 mm l-Phe, or 10 mm l-Phe and 1 μm NPS2143.HCl on food intake in rats with *ad libitum* access to food injected at the beginning of dark phase at 0–1 h post administration (*n*=18, crossover). (**c**) The effect of 50 mm l-Phe in presence or absence of 10 μm NPS2143.HCl on GLP-1 release from STC-1 cells following 2 h incubation, *n*=8 independent plates. (**d**) The effect of 30 mm l-Phe in presence or absence of 15 μm NPS2390 on GLP-1 release from primary mice colonic L cells following 2 h incubation, *n*=5 independent plates. (**e**) The effect of 30 mm l-Phe in presence or absence of 15 μm NPS2390 on GLP-1 release from mouse ileal organoids following 24 h incubation, *n*=8, L-Phe; 9, NPS2390, control; 10, L-Phe+NPS2390, independent organoid cultures. **P<0.01, ***P<0.001, ****P<0.0001 vs control; ^##^*P*<0.01, ^###^*P*<0.001 vs L-Phe. All data presented as mean±s.e.m.

l-Phe is a potent agonist of the CaSR,^32^ which has previously been implicated in amino-acid-induced gut hormone release *in vitro*.^7, 22^ CaSR is highly expressed and co-localised within GLP-1 and PYY secreting L cells.^19^ The expression of CaSR was detected in all levels of the GI tract in mice (Supplementary Figure S1). We hypothesised that the anorectic effect of l-Phe may be mediated via the CaSR. Oral administration of the synthetic CaSR agonist R568.HCl significantly reduced food intake during 0–1 h compared to the vehicle (*P*<0.05, *n*=8, vehicle; 9, R568; Figure 5a). Given that it was unclear where oral l-Phe was acting in the GI tract, and that high doses of a CaSR antagonist might affect systemic calcium homeostasis, we investigated the ability of a low dose of the CaSR antagonist NPS2143.HCl to block the anorectic effects of low dose intra-ileal l-Phe. The anorectic effect of intra-ileal administration of l-Phe was attenuated by the co-administration of NPS2143.HCl (*P*<0.05, *n*=18, crossover; Figure 5b). In addition, l-Phe-induced GLP-1 release from a murine primary colonic crypt culture was not significant in the presence of a CaSR antagonist, and l-Phe-induced GLP-1 release from STC-1 cells and murine ileal organoids was significantly attenuated by CaSR antagonists (Figures 5c–e).

### Repeated l-Phe administration reduces food intake and body weight in mice

**Figure 6:**
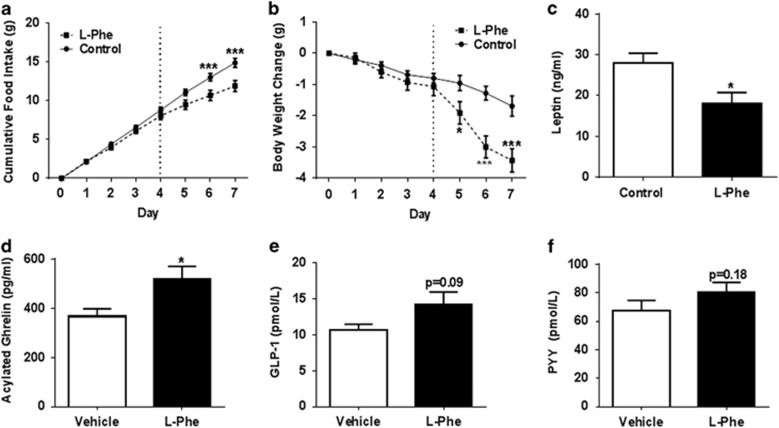
The effect of repeated OG administration of l-Phe on food intake, body weight and gut hormones in DIO mice. The effect of repeated OG of vehicle (10% TWEEN20 in water) or 12 mmol kg^−1^ l-Phe on (**a**) cumulative food intake, (**b**) body weight change, (**c**) plasma leptin, (**d**) acylated ghrelin, (**e**) GLP-1 and (**f**) PYY following twice daily OG during days 0–3 and three times daily OG during 4–7 days of chronic study. Dotted line highlights the start of three times daily injection. Plasma gut hormones and leptin levels were measured 24 h following the completion of the chronic administration in *ad libitum*-fed DIO mice. *n*=10 per group. *P<0.05, ***P<0.001 vs vehicle. All data expressed as mean±s.e.m.

We investigated whether the anorectic effects of l-Phe could be sustained chronically and affect body weight in DIO mice, a widely used model of obesity. Repeated twice daily oral administration of 12 mmol kg^−1^ l-Phe over 4 days had no significant effect on food intake or body weight in DIO mice. However, subsequent three times daily administration of 12 mmol kg^−1^ l-Phe significantly reduced cumulative food intake in DIO mice on days 6 and 7, and body weight on days 5, 6 and 7 (*n*=10; Figures 6a and b). Furthermore, repeated OG of 12 mmol kg^−1^ l-Phe significantly reduced plasma leptin levels in DIO mice compared to the controls (Figure 6c), suggesting that the reduced body weight reflected at least partly a loss of body fat. Chronic l-Phe administration also resulted in significantly increased plasma ghrelin levels (*P*<0.05, *n*=10; Figure 6d), and a trend towards increased plasma GLP-1 and PYY levels (GLP-1: *P*=0.1; PYY: *P*=0.19 vs controls; Figures 6e and f).

## Discussion

Evidence suggests that the satiety associated with high-protein diets may be mediated by sensing of the amino-acid constituents of protein, rather than by the sensing of the protein itself.^33^ l-Phe is an essential amino-acid abundant in dietary protein that activates the CaSR, a promiscuous amino-acid-sensing receptor expressed in the GI tract. The effects of l-Phe on appetite have been little studied, and the anorectic effects of intra-ileal administration of l-Phe on food intake in rodents have not previously been described.^34, 35^ Our studies demonstrate that l-Phe suppresses appetite and increases energy expenditure in rodents. Chronic l-Phe administration in DIO mice reduced food intake and body weight. l-Phe significantly stimulated GLP-1 release *in vitro* and *in vivo*, and improved glucose tolerance *in vivo*. Furthermore, we provide novel evidence of a role for the CaSR in the gut in the regulation of food intake as the anorectic effects of intra-ileal l-Phe were blocked by co-administration of a CaSR antagonist, and the l-Phe-stimulated release of GLP-1 from STC-1 cells was attenuated by a CaSR antagonist. Together these data provide evidence that pharmacological activation of the CaSR in the gut can modulate food intake, and suggest that the CaSR may have a role in sensing the amino-acid products of protein digestion to regulate appetite.

The anorectic effects of individual amino acids in rodents have recently been described.^35, 36^ Jordi *et al.*^35^ examined the effects of oral administration of amino acids, including l-Phe on food intake at a dose of 6.7 mmol kg^−1^ and found no significant effect on food intake. In contrast, our data demonstrate that oral administration of l-Phe at 3 mmol kg^−1^ robustly reduces food intake in fasted rats, and at doses between 6 and 12 mmol kg^−1^ reduces food intake in mice in the early light phase, and in *ad libitum-*fed rats and mice in the early dark phase. Mice required a relatively higher dose (12 mmol kg^−1^) of l-Phe to achieve a comparable reduction in food intake to rats. The doses of l-Phe orally administered in our feeding studies are pharmacological, but similar to the amount of l-Phe ingested daily by a rodent on a 45% protein diet. The anorectic effect observed is beyond that attributable to the calorific value of the given l-Phe solution. For example, a dose of 6 mmol kg^−1^ l-Phe is equivalent to ~1.54 kcal per rat, but resulted in a 4.81 kcal reduction in food intake; the observed reduction is still significant if these additional kilocalories are added to the energy intake of the treatment group. Oral l-Phe administration increased energy expenditure in rats, an effect that may be driven by increased locomotor activity, though the increase in locomotor activity observed did not itself achieve statistical significance.

Oral administration of the CaSR agonist R568.HCl significantly reduced food intake in rats, and direct ileal administration of 10 mm l-Phe in conscious rats significantly reduced food intake when injected in the dark phase, an effect that was attenuated by co-administration of the CaSR antagonist NPS.2143.HCl. This concentration of l-Phe has previously been demonstrated to stimulate the release of gut hormones from isolated rat small intestinal preparations, an effect also attenuated by a CaSR antagonist.^22^ These observations suggest a role for the CaSR in mediating the effects of l-Phe on food intake.

Previous studies have demonstrated that l-Phe can stimulate CCK release,^20, 21, 37^ and the release of PYY and GLP-1 from isolated loops of rat small intestine.^22^ Furthermore, *in vitro* evidence has suggested that the l-Phe-induced CCK release from STC-1 cells and the l-Phe-induced GLP-1 and PYY release from isolated small intestine are mediated by the CaSR.^20, 38^ In accord with these observations, treating STC-1 cells or ileal organoids with a CaSR antagonist attenuated l-Phe-induced GLP-1 release from STC-1 cells. However, treatment of cells with the antagonist resulted in only a partial reduction in GLP-1 release, suggesting that other mechanisms may also be involved in l-Phe-induced GLP-1 release. l-Phe can also activate the T1R1–T1R3 receptor,^5^ and a recent study demonstrated that l-Phe-stimulated CCK release from STC-1 cells requires the activity of the T1R1–T1R3 receptor.^7^ However, our findings suggest that l-Phe mediates its effects on gut hormone release at least in part via the CaSR.

Oral l-Phe reduced acylated ghrelin levels in rats. We have previously shown that l-cysteine reduces food intake in rats, an effect that may in part be due to a reduction in acylated ghrelin levels observed following oral administration.^35^ The CaSR is enriched in ghrelin cells of the stomach and CaSR activation by calcium chloride and R568.HCl inhibited ghrelin release from primary gastric mucosal cells *in vitro*.^38^ Interestingly, chronic l-Phe administration significantly increased circulating ghrelin levels. Plasma ghrelin levels are reduced in obese individuals and elevated following weight loss, an effect thought to represent a feedback loop intended to increase food intake when body weight drops.^39, 40^ The increase in ghrelin observed thus likely reflects the reduced body weight of these animals, rather than a specific effect of l-Phe. However, this increase in ghrelin does suggest that the reduction in food intake and the consequent weight loss are unlikely to be driven by changes in ghrelin signalling. Further studies investigating the effects of repeated administration of l-Phe over a longer time period are required to determine whether tolerance develops to its effects and to further assess the likely utility of such an approach to treat obesity.

Oral administration of l-Phe in rats significantly elevated circulating GLP-1 levels, and raised PYY levels, though this effect did not achieve statistical significance. l-Phe has previously been shown to stimulate GLP-1 release from murine primary colonic L cells.^41^ Direct administration of l-Phe into the ileum increased plasma PYY in rats, though this effect did not reach statistical significance (*P*=0.06). Interestingly, direct ileal administration did not alter plasma GLP-1 levels (data not shown). In humans, CaSR is highly expressed in the distal small intestine and a significant population of L cells express CaSR in the proximal colon. GLP-1 is expressed throughout the GI tract, whereas PYY-expressing cells are relatively scarce in the upper small intestine, and much more common in the distal small intestine and colon.^19^ Oral administration of l-Phe may stimulate GLP-1 release from enteroendocrine cells in the proximal gut, whereas direct ileal administration may stimulate PYY-secreting L cells. l-Phe stimulated the release of GLP-1 and PYY from a murine primary colonic L-cell culture, and of GLP-1 from STC-1 cells. It is therefore interesting that direct ileal administration did not influence circulating GLP-1 levels. Recent evidence suggests that GLP-1 and PYY are packaged in different secretory granules within L cells, raising the possibility that the release of these hormones can be differentially regulated.^42^ Alternatively, only specific PYY-expressing populations of L cells in the ileum may respond to l-Phe.

l-Phe has previously been found to stimulate CCK release both *in vitro*^20^ and *in vivo*,^43, 44^ but we did not observe this effect in our studies. However, previous studies either used considerably higher doses or concentrations of l-Phe, or the l-Phe was administered in the form of a di-peptide. It is also possible that the single 30 min time point we used missed an effect on CCK release. Chronic l-Phe administration in DIO mice did not result in statistically significant differences in circulating GLP-1 or PYY levels, though concentrations were higher than that in vehicle-treated controls. This may reflect that the animals had not received l-Phe for 24 h; l-Phe may largely have acute effects on the release of these hormones.

Specific amino acids are known to influence glucose homeostasis. l-Arginine stimulates the release of insulin from pancreatic β-cells;^45, 46^ and l-glutamine, a GLP-1 secretagogue *in vitro*, stimulates insulin and improves glucose homeostasis in obese and diabetic human subjects.^47, 48^ Given that oral l-Phe elevated plasma GLP-1 levels in rats, we hypothesised that it would also influence glucose homeostasis. In accord with this, oral l-Phe significantly increased insulin release and improved glucose tolerance in response to an IPGTT in rats. Further work is required to identify whether this effect is exclusively mediated by the effects of l-Phe on GLP-1 release; CaSR is also expressed on pancreatic beta cells,^49^ and the effects observed feasibly reflect direct effects on insulin release. A recent study has demonstrated improved glucose tolerance in rats following activation of the gut CaSR system by synthetic peptide agonists.^50^

In summary, our data suggest that sensing of l-Phe in the GI tract can modulate hormone release, glucose tolerance and appetite, and demonstrate for the first time that l-Phe can act via the CaSR to reduce food intake *in vivo*. Though it has been speculated that promiscuous G-protein-coupled amino-acid receptors in the gut may regulate feeding behaviour, our study represents the first time that amino-acid sensing by such a receptor in the GI tract has been demonstrated to influence food intake. While pharmacological, our findings may represent activation of a physiological nutrient sensing system. Further work is required to determine the role of the CaSR in the sensing of dietary protein, and the utility of exploiting l-Phe and the GI CaSR system in the treatment of obesity and diabetes.
