# Supplementary material for: l-phenylalanine modulates gut hormone release and glucose tolerance, and suppresses food intake through the calcium-sensing receptor in rodents
Source: Int J Obes (Lond). 2017 Aug 8;41(11):1693–701. doi: 10.1038/ijo.2017.164 (PMC5678004; doi:10.1038/ijo.2017.164)
Supplement: Supplementary Materials [file ijo2017164x1.docx]

**Online Supplemental Materials.**

**L-phenylalanine stimulates gut hormone release and improves glucose tolerance, and suppresses food intake by activating the calcium sensing receptor in rodents.**

Amin Alamshah^1^, Eleanor Spreckley^1^, Mariana Norton^1^, James S. Kinsey-Jones^1^, Anjali Amin^1^, Rebecca Johnson^1^, Keenan Saleh^1^, Elina Akalestou^1^, Zainab Malik^1^, Ahmad Moolla^1,2^, Peter R. Sargent^3^, Gary W. Gray^3^, Stephen R. Bloom^1^, Kevin G. Murphy^1^

^1^Section of Endocrinology and Investigative Medicine, Department of Medicine, Imperial College London, London, UK.

^2^Oxford Centre for Diabetes, Endocrinology and Metabolism, University of Oxford, Oxford, UK

^3^TasteTech Ltd, Bristol, UK.

**Supplementary Figure 1. The expression of CaSR in the GI tract in mice.**


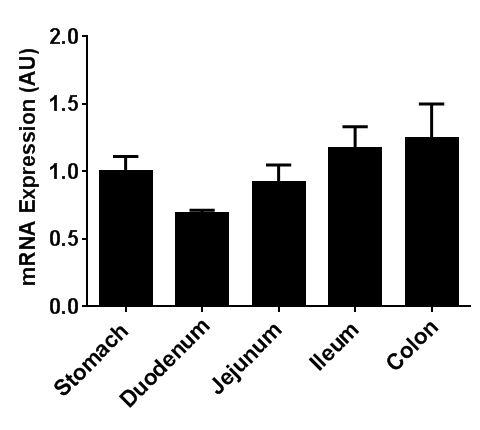


Figure 1. The expression of CaSR in the GI tract in mice. Relative expression of CaSR mRNA in different regions of the GI tract in overnight fasted male mice. Mice were fasted for 16 hours overnight to avoid any effects of acute food intake on gene expression, then decapitated, and the stomach, duodenum, jejunum, ileum and colon rapidly removed, snap frozen in liquid nitrogen and stored at -80°C for RNA extraction and real-time quantitative PCR (qPCR) analysis. Total RNA was extracted from tissues using TRI reagent (Sigma, Poole, UK) according to the manufacturer's instructions. High Capacity cDNA Reverse Transcription Kit (Life technologies, Paisley, UK) was used to generate cDNA template for qPCR according to the manufacturer's instructions. qPCR was performed using TaqMan Gene Expression Assays and TaqMan Universal PCR Master Mix (Life technologies, Paisley, UK) using the ABI Prism 7900 Sequence Detection System according to the protocols provided by the manufacturer (Life technologies, Paisley, UK). The relative mRNA transcript levels were calculated according to the 2^–ΔCT^ method, with ΔCT being the difference in cycle threshold values between the CaSR mRNA (mCG130161), and the hypoxanthine phosphoribosyltransferase 1 mRNA (m00446968_m1) internal control. Expression levels are shown as relative to stomach expression. n= 4-6 per region.
